# Diversity of bacteriome associated with *Phlebotomus chinensis* (Diptera: Psychodidae) sand flies in two wild populations from China

**DOI:** 10.1038/srep36406

**Published:** 2016-11-07

**Authors:** Kaili Li, Huiying Chen, Jinjin Jiang, Xiangyu Li, Jiannong Xu, Yajun Ma

**Affiliations:** 1 Department of Tropical Infectious Diseases, Faculty of Tropical Medicine and Public Health, Second Military Medical University, Shanghai 200433, China; 2 Biology Department, Molecular Biology Program, New Mexico State University, Las Cruces NM 88003, USA

## Abstract

Sand fly *Phlebotomus chinensis* is a primary vector of transmission of visceral leishmaniasis in China. The sand flies have adapted to various ecological niches in distinct ecosystems. Characterization of the microbial structure and function will greatly facilitate the understanding of the sand fly ecology, which would provide critical information for developing intervention strategy for sand fly control. In this study we compared the bacterial composition between two populations of *Ph. chinensis* from Henan and Sichuan, China. The phylotypes were taxonomically assigned to 29 genera of 19 families in 9 classes of 5 phyla. The core bacteria include *Pseudomonas* and enterobacteria, both are shared in the sand flies in the two regions. Interestingly, the endosymbionts *Wolbachia* and *Rickettsia* were detected only in Henan, while the *Rickettsiella* and *Diplorickettsia* only in Sichuan. The intracellular bacteria *Rickettsia, Rickettsiella* and *Diplorickettsia* were reported for the first time in sand flies. The influence of sex and feeding status on the microbial structure was also detected in the two populations. The findings suggest that the ecological diversity of sand fly in Sichuan and Henan may contribute to shaping the structure of associated microbiota. The structural classification paves the way to function characterization of the sand fly associated microbiome.

Insects are intimately associated with a symbiotic. The ecological interactions between the microflora and host have a profound impact on various host life traits^1^,^2^,^3^,^4^,^5^. Characterization of the structure and function of host associated microbiome is essential for a comprehensive understanding of the biology and ecology of host insect. Hematophagous phlebotomine sand flies (Diptera: Psychodidae: Phlebotominae) are the insect vectors responsible for the transmission of protozoan *Leishmania* species (Euglenozoa: Trypanosomatidae), the causative parasites of the leishmaniasis^6^. As a critical aspect of sand fly ecology, surveys on microflora have been conducted for sand flies *Phlebotomus papatasi*, *Ph. argentipe*, *Ph. sergenti, Ph. kandelakii, Ph. perfiliewi* and *Lutzomyia longipalpis*^7^,^8^,^9^,^10^,^11^,^12^,^13^,^14^,^15^,^16^. Regarding the microbial contributions to the physiology of sand flies, gut bacteria have been shown to play roles in development^17^ and immunity^18^,^19^. In addition, evidence has become available that resident microorganisms in the gut are able to inhibit *Leishmania* infections^20^,^21^.

*Phlebotomus chinensis* is a principal vector for the visceral leishmaniasis (VL) in China with a wide geographic distribution^22^. Recently, we have developed molecular markers for the *Ph. chinensis* identification and confirmed the species discrimination between *Ph. chinensis* and *Ph. sichuanensis*^23^,^24^. The population structure of *Ph. chinensis* was investigated using microsatellite markers as well^24^. In China, the mountainous areas in Sichuan Province are the endemic foci of mountain type zoonotic VL, where dogs and wild animals are served as reservoir hosts. The hilly ground areas in Henan Province represent the endemic foci of anthroponotic type of VL, where humans are major reservoirs^25^. The biotic and abiotic conditions between the two geographic environments are quite different. The ecological aspects of *Ph. chinensis* in the two regions differ in terms of sand fly habitats, types of human dwellings and vegetation coverages. Understanding of sand fly ecology is essential to the development of appropriate fly control strategies. Recently we reported the ecological niches and blood sources of *Ph. chinensis* in Sichuan^26^. In this paper, we present the bacterial composition and diversity in wild *Ph. chinensis* collected from Sichuan and Henan, China, which were characterized by using both culture independent and dependent approaches.

## Results

### Sand fly collections and identification of sand fly species

Sand fly specimens were collected in Henan (HN) and Sichuan (SC), China. A total of 1061 specimens were used in this study. In Henan collection, there were 200 males (HNM), 200 females (HNF) and 90 blood-fed females (HNB). In Sichuan collection, there were 241 males (SCM), 240 females (SCF) and 90 blood-fed females (SCB). All specimens were identified as *Ph. chinensis* by morphology and confirmed by sequencing a species-specific ribosomal DNA-Internal Transcribed Spacer 2 PCR fragment.

### Taxonomic diversity of bacteria in Henan and Sichuan samples

The bacterial 16S ribosomal DNA fragment was amplified from genomic DNA by universal primers, and the PCR products were cloned into the plasmid TOPO-TA. A total of 1251 clones were sequenced and analysed. The sequences were classified using a classifier at Ribosomal Database Project at Michigan State University^27^. The taxonomic composition of different sand fly pools from the two regions was presented in Table S1. The phylotypes were classified into 5 phyla, 9 classes, 17 orders, 19 families and 29 genera, with a few unclassified taxa at different ranks. Most phylotypes were placed in Classes α- and γ-Proteobacteria, and Bacilli. In Henan collection, 23 genera were recognized in blood-fed flies (HNB), 14 in females (HNF) and 9 in males (HNM); while in Sichuan collection, 14 genera were found in blood-fed females (SCB), 9 in females (SCF) and 9 in males (SCM). Overall, more taxa were present in blood-fed specimens than in blood-unfed females and males.

**Figure 1:**
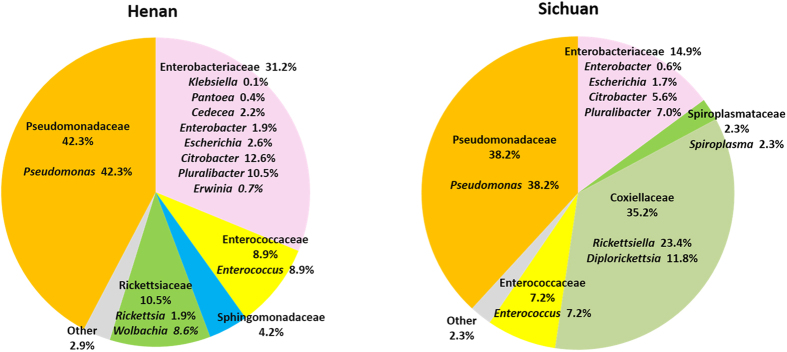
The taxonomic composition and abundance of bacteria in the sand flies from Henan and Sichuan collections. The pies represent taxa at family level; the genus was presented in italic within families.

The taxonomic composition between the two collections was further compared at family and genus level (Fig. 1). In α-Proterobacteria, 9 genera were recognized in Henan and 6 in Sichuan. Interestingly, two obligate intracellular bacteria, *Rickettsia* (1.9%) and *Wolbachia* (8.6%), were found only in Henan collection, while intracellular bacteria *Diplorickettsia* and *Rickettsiella* (35.3%) and *Spiroplasma* (2.3%) were only detected in Sichuan samples. In γ-Proteobacteria, families Enterobacteriaceae, Pseudomonadaceae and Coxiellaceae were dominant taxa. Enterobacteriaceae counted for 31.2% in Henan collection and 14.9% in Sichuan collection. In family Pseudomonadaceae, the phylotypes were all assigned to genus *Pseudomonas*. The abundance of *Pseudomonas* was similar between the two locations, 42.3% in Henan and 38.3% in Sichuan. In Firmicutes, family Enterococcaceae was dominated by *Enterococcus*, which had similar abundance between the two collections, 8.9% in Henan and 7.2% in Sichuan.

**Figure 2:**
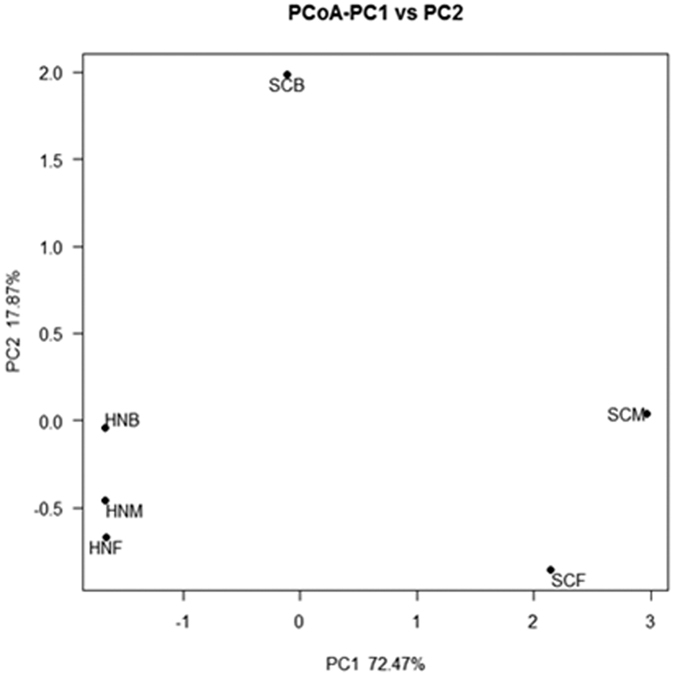
PCoA plot of bacterial diversity in the sand fly samples from Henan and Sichuan collections.

The microbial structure between the two collections was visualized by a Principal Coordinate Analysis (PCoA). The PCoA plot revealed that the microbial communities from Henan samples were close to each other, while those from Sichuan samples were discrete (Fig. 2). The pattern suggested that microbial structure in the Henan samples was similar, but the structure was quite diverse among the Sichuan samples.

In addition to the 16S rDNA PCR based bacterial profiling, a culture dependent bacterial isolation was conducted as well. A total of 21 colonies were obtained, 8 phylotypes were recognized (Table S2). Among them, 5 belong to *Pseudomonas*; and the other phylotypes include the taxa that are close to *Acinetobacter junii*, *Bacillus thuringiensis* and *Staphylococcus warneri*.

### Phylogeny of taxa in genera *Pseudomonas, Diplorickettsie* and *Rickettiaceae*

**Figure 3:**
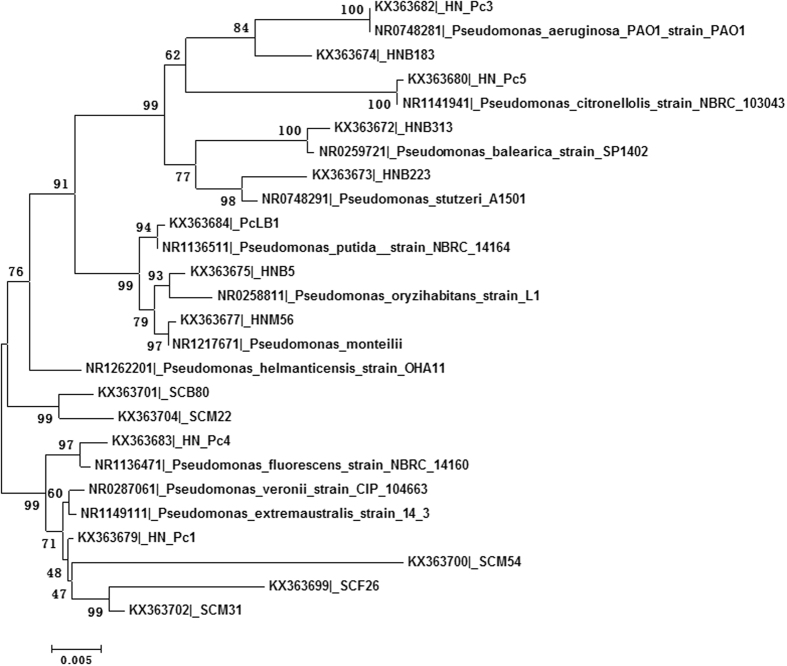
The phylogenetic relationship of the known *Pseudomonas* and related phylotypes from *Ph. chinensis* inferred by neighbour-joining method. Bootstrap values were placed on the nodes.

A total of 521 sequences were assigned to genus *Pseudomonas*. When these sequences were aligned, 508 were grouped into 5 consensus sequences, and 10 singletons remained. These 15 phylotypes showed similarity to 11 species of *Pseudomonas* spp. The phylogenetic affiliation of these sequences was present in Fig. 3. The sequences formed two clades, 8 known species (*P. citronellolis, P. aeruginosa, P. stutzeri, P. balearica, P. monteilii, P. oryzihabitans, P. putida*, and *P. helmanticensis*) and 3 consensus (Pc3/KX363682, Pc5/KX363680, PcLB1/KX363684) and 7 singleton sequences clustered together into one clade, and 3 known species (*P. fluorescens, P. extremaustralis*, and *P. veronii*) and 2 consensus (Pc4/KX363683, Pc1/KX363679) and 3 singleton sequences formed a separate clade. The phylotype sequences have various degree of distance to the known species. Evidently, there are novel *Pseudomonas* spp. that are associated with the sand flies.

**Figure 4:**
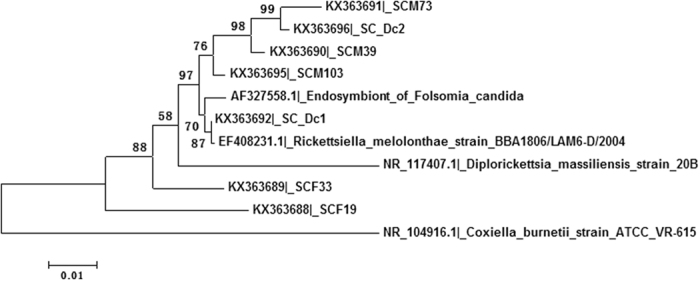
The phylogenetic relationship of the known taxa in family Coxiellaceae and related phylotypes from *Ph. chinensis* inferred by neighbour-joining method. Bootstrap values were placed on the nodes.

The phylotypes that are closely related to *Diplorickettsia* were only detected in Sichuan collection. A total of 183 sequences were similar to *Diplorickettsia*, which were represented by 2 consensus sequences and 5 singletons. Consensus 1 and 2 contained 124 and 54 sequences, respectively. The sequences had certain similarity with intracellular bacteria, *Rickettsiella melolonthae, Diplorickettsia massiliensis* and *Coxiella burnetii*. The phylogenetic tree showed that consensus 1 (Dc1/KX363692) was closely related to *R. melolonthae* and formed a clade with another endosymbiont sequence (GenBank accession AF327558). The consensus 2 (Dc2/KX363696) and other three singletons grouped into a sister clade, both clades were internal to *D. massiliensis*, a bacterial species associated with *Ixodes ricinus* ticks^28^. The other two singletons represent novel taxa in the family (Fig. 4).

Family Rickettsiaceae was only found in Henan specimens. All of 76 sequences were grouped into two consensus sequences and one singleton. Consensus 1 consists of 13 sequences, which hit to *Rickettsia bellii*, and consensus 2 contains 62 sequences, which hit to *Wolbachia* sp. wRi. Both hits have 100 coverage and 99% identity.

## Discussion

In this study we used 16S rDNA primers 27F and 1492R to amplify an approximately 1450 bp rDNA fragment. The length of the amplicon is close to the full length of ribosomal RNA gene, which provides almost complete sequence information for comparison to the references in the bacterial 16S rDNA database. Therefore, the accuracy of the taxonomic classification of these sequences is much better than the short reads generated by high throughput sequencing methods, such as pyrosequencing and Illumina sequencing. However, due to the low throughput, the numbers of sequences may not be sufficient to provide a comprehensive estimate of complete microbial composition, low abundant taxa could be missed. In this study, 1251 sequences were assigned into 29 genera in 5 phyla. At genus level, novel phylotypes were identified, which were closely related to but not identical to known species. We intend to believe that the methods have detected the phylotypes that are relatively abundant and PCR amplifiable, as exemplified by the phylotypes in *Pseudomonas* (Fig. 3) and the intracellular phylotypes in family Coxiellaceae (Figs 1 and 4).

The data showed that the taxa in families Pseudomonadaceae, Enterobacteriaceae and Enterococcaceae were predominant in the sand fly samples from both Henan (82.4%) and Sichuan (60.3%) (Fig. 1). Diverse taxa in *Pseudomonas* were present in both Henan and Sichuan specimens. The diversity and abundance of the phylotypes in family Enterobacteriaceae were enriched in the blood-fed specimens from both Henan and Sichuan. A similar composition shift has been found in sand fly^8^ and mosquito gut microbiota^29^. Intracellular bacteria appear to be distinct in the two geographic collections. *Wolbachia* and *Rickettsia* were found only in Henan samples, and *Diplorickettsia* and *Spiroplasma* were found only in Sichuan samples. The microbial diversity among three pools (male, female and blood-fed female) in both Henan and Sichuan were visualized by the PCoA plot (Fig. 2). There were detectable differences among the three pools. The patterns were similar in Henan collection but diverse in Sichuan collection, which suggested that sex and feeding status had a stronger impact on the microbial structure in Sichuan than in Henan samples. Likely, the difference may attribute to the distinctive geographic environments and diverse ecological niches of sand flies between Sichuan and Henan. In Sichuan, the sand flies were sampled in a rock cave and a rabbit farm in a village along a mountain valley, and sand flies there prefer to take blood from pigs, dogs and chickens^26^. In Henan, the sand flies were sampled in a village where human dwellings were cave houses carved out of the side of cliffs. The sand flies were caught in human dwellings and animal pens. The blood source identification revealed that the sand flies in the village took blood from humans and various domestic animals including dogs, chickens, pigs and cows (unpublished data). The plant composition and coverage are quite different between the two regions, which has a significant impact on the animal fauna as well. Different ecological characters may have a significant impact on sand flies and their associated microbes. It has been shown that the acquisition of mosquito microbiota is associated with the eco-environment where mosquitoes inhabit^30^. Therefore, it is reasonable to speculate that the different microbial structure between the two sand fly samples from Henan and Sichuan may result from the interactions of the sand flies and their habitats. Due to the limited sample collections and the less comprehensive methodology used in this study, we did not intend to address questions regarding the connections among host traits, microbial structure and environmental parameters. More sample collections from wider geographic regions are needed to better characterize the microbial ecology of the sand fly associated microbiota.

*Pseudomonas* spp. are commonly found in sand flies^7^,^8^,^10^,^11^,^31^. In this study, the phylotypes similar to *Pseudomonas* were grouped into two clades with 11 known species (Fig. 3), which suggests the presence of novel taxa in the *Pseudomonas* associated with the sand flies. However, the 16S rDNA sequences do not have sufficient resolution to infer the relationships among these taxa, as the bootstrap values were less than 60 in several nodes within the clade. It is well known that 16S rDNA has poor resolution at the intra-genus level for *Pseudomonas*^32^,^33^. Currently, multilocus sequence analysis (MLSA) and comparative genomics approach have become necessary to build a phylogenomic taxonomy for *Pseudomonas*^34^. Therefore, the true identity of these phylotypes needs further study using additional bacteriological and genomic methods. Interestingly, in *L. longipalpis*, an isolate of *Pseudomonas*, *Pseudomonas* sp. KK-1, has been shown to support larval development and to be able to pass through pupation and remain in the digestive tract of newly emerged females^17^. Taxa in Enterobacteriaceae are commonly associated with sand flies, such as *Serratia, Enterobacter, Pantoea, Klebsiella*, and *Citrobacter*^7^,^8^,^11^,^12^,^14^,^17^,^31^. In this study, *Citrobacter* and *Pluralibacter* were found with high frequency in both Sichuna and Henan specimens. Genus *Pluralibacter* was recently separated from *Enterobacter*^35^. Duo to the dominance of various taxa of *Pseudomonas* and enterobacteria in sand flies, further studies are needed to investigate their contributions to the life traits of sand flies.

Intracellular bacteria, such as *Wolbachia*, have been found in *Lutzomyia* and *Phlebotomus* sand flies^36^,^37^,^38^. *Spiroplasma* as endosymbionts has been identified in *Drosophila*^39^,^40^,^41^,^42^, ticks^43^,^44^ and mosquitoes^45^. *Diplorickettsia*, *Coxiella* and *Rickettsiella* are obligate endosymbionts that have been identified in ticks^28^,^46^,^47^,^48^ and leafhoppers^49^. Here, we reported for the first time the presence of *Spiroplasma* and taxa related to *Diplorickettsia* and *Rickettsiella* in *Ph. chinensis* sand flies. Endosymbionts have complex interactions with their hosts, such as parthenogenesis, male killing, cytoplasm incompatibility, and host defence^41^,^50^,^51^,^52^,^53^. Although most of *Rickettsiella* spp. in arthropods have been thought to be pathogenic, a nonpathogenic *Rickettsiella* spp. found in *Acyrthosiphon pisum* pea aphids has been shown to be evolutionarily beneficial to the aphids by changing the body colour to reduce predation^54^. Little knowledge of the interactions between intracellular bacteria and sand flies are available, which warrants further studies in order to understand the ecology of sand flies and associated microorganisms.

In this study, we also obtained 8 bacterial strains from culture. Recently, the genomes of several mosquito derived bacterial strains have been sequences, including *Asaia* sp., *Elizabethkingia anophelis*, *Enterobacter* sp., *Pseudomonas* sp., *Serratia* sp., *Stenotrophomonas maltophila*, and *Staphylococcus hominis*^55^,^56^,^57^,^58^,^59^,^60^,^61^. These genome data allow one to analyse the microbial genetic repertoire that may be associated with symbiosis. The availability of bacterial strains derived from this study enables further characterization of bacterial genetic capacity and comparison between host associated and non-associated bacterial strains. Such comparison will generate insights into the microbial roles in the symbiotic interactions with the hosts.

## Conclusions

This survey revealed a complex microbial structure in *Ph. chinensis* from two distinct ecosystems in Henan and Sichuan, China. Sand flies have adapted to various ecological niches with different terrestrial vegetation. They are able to thrive in various types of habitats and take blood from a variety of animal sources. Such eco-adaptability has an influence on the structure of associated microbial communities. Core taxa include *Pseudomonas* and enterobacteria. Intracellular bacteria from 4 genera were identified, among which *Diplorickettsia*, *Rickettsiella* and *Spiroplasma* were reported for the first time in sand flies.

## Materials and Methods

### Ethical statement

This study was carried out in strict accordance with the NSFC, NIH and NMSU ethical guidelines for biomedical research.

### Sand fly collection and species identification

The specimens of sand flies were collected in the field trips in Shanxian County, Henan Province, China, in June 2015, and Jiuzhaigou Country, Sichuan province, China, in July 2015. The GPS coordinates of the site were 110.10°E/34.24°N, altitude 985 m in Henan, and 104.25°E/33.24°N, 1506 m in Sichuan. The CDC mini light traps (BioQuip, USA) and light traps (Shengzhen, China) were used for catching sand flies. With the owners’ consent, the light traps were installed between 6:30 pm-8:30 am in chicken houses and courtyards in Henan. In Sichuan, the light traps were set up in a rabbit farm and a rock cave as described previously^26^. Specimens of *Ph. chinensis* were sorted out by morphological keys^62^ and confirmed by sequencing the ribosomal DNA PCR products as we described previously^23^. Specimens were separated into three groups: males, females and blood-fed females (with visible blood residues in the specimens). Individuals were surface cleaned by dipping consecutively in three tubes with 75% ethanol. Henan samples included 200 males, 200 females and 90 blood-fed females. Sichuan samples included 241 males, 240 females and 90 blood-fed females. About 20 sand flies were pooled together and preserved in RNAfixer (Aidlab Biotechnologies Co., Ltd, China) until DNA extraction. The DNA was isolated using DNAzol (Life Technologies, USA), following the manufacturer’s instruction.

### Bacterial 16S ribosomal gene PCR

The primers 27F (5′-GAG TTT GAT CCT GGC TCA G-3′) and 1492R (5′-TAC GGC TAC CTT GTT ACG ACT T-3′)^63^ were used to amplify 16S ribosomal RNA gene fragment. The PCR reaction was run in a 25 μl mix including 1.0 μl DNA template, 0.2 μM primers, and other PCR reagents (Aidlab Biotechnologies Co., Ltd, China). The parameters were set as 94 °C for 30 s, 52 °C for 30 s, 72 °C for 30 s, for 35 cycles. The PCR products were purified and cloned into the TOPO-TA plasmid (Life Technologies, USA) following the manufacturer’s instruction. The inserts were sequenced for both strands using M13 forward and reserve primers at Boshang Biotech Co., Ltd (Shanghai, China). The sequences have been submitted to NCBI, which are accessible under GenBank accession numbers KX363666- KX363706.

### Bacterial culture

For the bacterial culture purpose, 10 males and 10 females were surface cleaned in 75% ethanol and pooled together, respectively. The pooled specimens were stored in Luria-Bertani (LB) medium and brought back to the laboratory. The specimens were homogenized in LB medium. Diluted homogenates were spread onto a LB agar plate, which was incubated at room temperature until colonies were grown. Single colonies were transferred to liquid LB to culture. The bacterial identity was determined by 16S rDNA PCR and sequencing as described above. Unfortunately, there were not enough blood fed specimens in the collections. So no blood fed specimens were used for culture.

### Taxonomic identification and phylogenetic analysis

The sequences were inspected and aligned at 97% of similarity using SeqMan program (DNAstar, CA, USA)^64^. The sequences with 97% identities were grouped into a contig. Consensus sequences of the contigs and singleton sequences were classified using a RDP classifier^27^ at https://rdp.cme.msu.edu/classifier/classifier.jsp. The sequences were further compared using the blastn search tool against 16S ribosomal RNA sequences (Bacteria and Archaea) and Nucleotide collection (nr/nt) databases at NCBI. The taxon identity of phylotypes and frequency of each phylotype were compared between different samples. MEGA6 software^65^ was used for phylogenetic analysis. The phylotype sequences and related homologues from GenBank were aligned using Clustal W. Maximum Likelihood (ML) and Neighbour-Joining (NJ) methods with different parameters were tested for phylogeny. Before ML tree construction, the best DNA model were found as the *HKY + G + I* model. The NJ trees were constructed using *p* distance and 1000 bootstrap replications. Both methods generated very similar results. So only NJ trees were presented.

### Principal Coordinate Analysis

The principal coordinate analysis was used to visualize the variance associated with microbial structure in different samples, based on weighted unifrac and unweighted unifrac distance matrix, implemented in the software QIIME (Quantitative Insights Into Microbial Ecology, http://qiime.org/).

## Additional Information

**How to cite this article**: Li, K. *et al*. Diversity of bacteriome associated with *Phlebotomus chinensis* (Diptera: Psychodidae) sand flies in two wild populations from China. *Sci. Rep*. **6**, 36406; doi: 10.1038/srep36406 (2016).

**Publisher’s note:** Springer Nature remains neutral with regard to jurisdictional claims in published maps and institutional affiliations.

## Supplementary Material

Supplementary Table S1

Supplementary Table S2
